# Evaluation of Color and Spectral Behavior of a Novel Flowable Resin Composite after Water Aging: An In Vitro Study

**DOI:** 10.3390/ma15124102

**Published:** 2022-06-09

**Authors:** Fei Chen, Di Wu, Rafiqul Islam, Yu Toida, Chiharu Kawamoto, Monica Yamauti, Hidehiko Sano

**Affiliations:** 1 Department of Stomatology, Beijing Tongren Hospital, Capital Medical University, Beijing 100176, China; 2 Department of Restorative Dentistry, Faculty of Dental Medicine, Hokkaido University, Kita 13, Nishi 7, Kita-ku, Sapporo 060-8586, Japan; wudi0526@den.hokudai.ac.jp (D.W.); rony12cdc@den.hokudai.ac.jp (R.I.); rinnum@den.hokudai.ac.jp (Y.T.); chipa@den.hokudai.ac.jp (C.K.); myamauti@den.hokudai.ac.jp (M.Y.); sano@den.hokudai.ac.jp (H.S.)

**Keywords:** tooth color, flowable resin composite, color stability, structural color, light transmittance, light reflection

## Abstract

Background: This study aimed to evaluate the color matching, light transmittance, and reflectance characteristics of the novel flowable resin composite OCF-001 (OCF). Methods: Fifty-four resin composite molds were made with simulated class I cavities of A2, A3, and A4 shades by filling the rubber mold interspace with Estelite Sigma Quick (ESQ), Gracefil Putty (GP) and Filtek Supremme Ultra (FSU). After applying the adhesive, three different flowable resin composites (*n* = 6), OCF, Gracefil LoFlo (GLF), and Supreme Ultra Flowable (SUF), were used to fill the cavities. A colorimeter was used to measure the color parameters (CIEDE2000). The color measurements were taken immediately and after 28 days. Data were analyzed using the nonparametric Kruskal–Wallis (α = 0.05) and Wilcoxon tests. The light transmittance and reflection characteristics were measured with a black background using a spectrophotometer under D65 illumination. Results: The ΔE_00_, and ΔC of OCF was lower than other tested materials in A2 and A3 shades both immediately and after 28 days. OCF showed the highest transmittance characteristic, and a relatively stable reflectance curve in all the wavelengths. Conclusions: OCF showed better shade matching with the surrounding shades of A2 and A3, a relative uniform reflectance and higher light transmission properties.

## 1. Introduction

Flowable resin composites (FRCs) have been very popular among clinicians since they were first introduced in 1996 [1]. The amount of inorganic filler content is lower in the FRCs, and the proportion of diluting monomer is increased compared with conventional resins. Consequently, the FRCs flow easily, thus favoring the accommodation of the material into difficult-to-access cavities [2,3,4]. In addition, because of their low viscosity, FRC materials can be used in minimally invasive preparations, and they can also be used as a sealant on the unprepared part of the occlusal surface [5].

Esthetics is always a significant concern for resin composites, including FRCs [6]. The esthetic success of color matching resins to restored teeth may be correlated to aspects of the restorative material’s optical properties, such as light transmission through the material, light reflection on its surface, light diffusion, absorption, and scattering [7,8]. In addition, the type and content of the inorganic filler and the organic matrix may be correlated to the optical properties, influencing the color matching ability [9]. It has been reported that FRCs with less filler content might exhibit different optical properties than paste-type resin composites [6,10].

Color stability remains a challenge, with changes observed immediately after polymerization and after some storage time [11,12]. FRCs with lower filler loading are related to lower color stability. Furthermore, the lower filler content would yield higher water sorption values, thus leading to the absorption of pigments and ions and to discoloration [13]. In addition to acting as a discoloring agent, water can cause color instability and variations in opacity. Because of the increased proportion of organic substances in the composite, the material would be more susceptible to water absorption and discoloration over time [14]. This has already been shown for microfilled composites with a higher organic content than microhybrid composites. For this reason, the color stability of FRCs should be lower because of the higher amount of resin matrix [15]. Incorporating color pigments into FRCs generally produces a clinically acceptable shade, affecting the color and diffusion of light through the composites.

Recently, a novel aesthetic flowable resin composite, OCF-001 (OCF), was developed, claiming to cover all VITA classical shades with a single shade [6]. The flowable character of OCF can be adapted to some small preparations with better sealing, thus filling the gaps for clinical needs. The composition of OCF is mainly based on a paste-type resin composite (Omnichroma). Neither contains pigment, and their uniformed spherical-shape filler of 260 nm would increase the reflection and refraction of a specific wavelength, which can be named the structural color technique [16]. In our previous study, the paste-type universal shade resin composite of Omnichroma showed a satisfactory esthetic quality based on its structural color, which comes from the physical interaction of regularly distributed supra-nano spherical fillers of the materials through light reflection and refraction [16]. However, the color measurement, light transmittance, and spectral reflectance of the OCF flowable resin composite have not been evaluated.

Therefore, the purposes of this study were (1) to determine the color matching of the flowable resin composite OCF using simulated class I cavities before and after water sorption and (2) to evaluate the light transmittance and reflectance characteristics of flowable resin composite OCF. The null hypotheses were that no significant differences would be detected in (1) the color matching and corresponding color parameters and (2) in the color stability of tested flowable resin composites.

## 2. Materials and Methods

### 2.1. Preparation of Specimen

A cured silicone impression material (Correcsil, Yamahachi Dental Mfg, Co., Aichi, Japan) was used to make a custom rubber mold with a round cavity (10 mm in diameter, 5 mm in height) and a small cylindrical projection (4 mm in diameter, 2 mm in height) in its center. Fifty-four resin composite molds were made for color measurement with simulated class I cavities of A2, A3, and A4 shades by filling the rubber mold interspace with Estelite Sigma Quick (ESQ, Tokuyama Dental, Tokyo, Japan), Gracefil Putty (GP, GC Corporation, Tokyo, Japan) and Filtek Supreme Ultra (FSU, 3M ESPE, St. Paul, MN, USA). An LED curing light (PenCure 2000, Morita, Tokyo, Japan) ranging from 660 to 760 mW/cm^2^ was used to cure the resin composites. According to the manufacturer’s instructions, a thin layer of chemical-cure adhesive (Bondmer Lightless, Tokuyama Dental, Tokyo, Japan) was applied to the cavity walls. After that, three different flowable resin composites (*n* = 6), Universal shade OCF-001 (OCF; Tokuyama Dental, Tokyo, Japan), Universal shade Gracefil LoFlo (GLF; GC Corporation, Tokyo, Japan), and A3B shade of Supreme Ultra Flowable (SUF; 3M ESPE, St. Paul, MN, USA), were used to fill the simulated class I cavities. The details of the materials used in this study are tabulated in Table 1. The materials were then light-cured according to the manufacturer’s instructions. Then, the restoration surface was polished with 1500-grit silicon carbide paper (Sankyo-Rikagaku, Saitama, Tokyo, Japan) and finished for 30 s each with fine and extra-fine finishing discs (SNAP, Shofu Inc, Tokyo, Japan). All of the specimens were aged artificially in distilled water at 37 °C, and their colors were measured immediately and after 28 days.

### 2.2. Color Measuring Procedure

A colorimeter (OFC-300, RC500, PaPaLaB Co., Tokyo, Japan) was used to measure the color parameters under D65 illumination, equivalent to “average” daylight. The equipment was calibrated using a white title background (L = 93.2; a = −0.3; and b = 1.6) and a black title background (L = 0.5; a = 0.7; and b = −0.6) immediately before each round of measurements. The reflectance measurements were performed under bi-directional 45/0° illumination. The measuring spot was selected at least 0.3 mm away from the margin, without crossing over the central flowable resin composite and peripheral resin composite molds [17], in order to assess the color parameters. The color measurement was repeated three times for the central and peripheral sides of each mold and the average was calculated.

**Figure 1:**
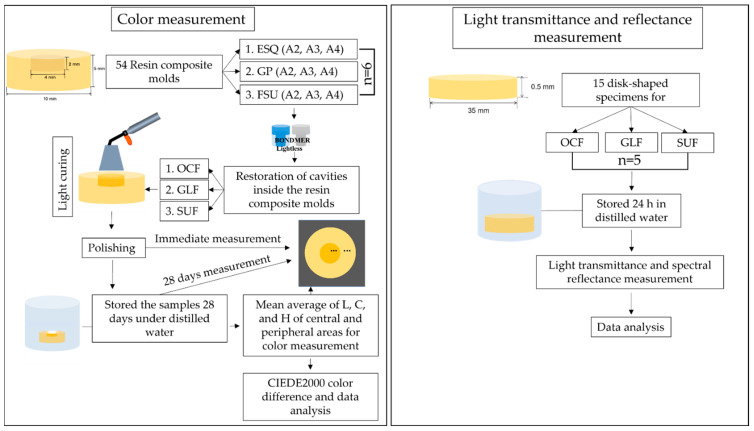
Illustrated flowchart of the preparation of specimens for color measurement, and for light transmittance and reflectance measurement.

An illustrated flowchart of the preparation of the specimen for color measurement is shown in Figure 1. CIE presently recommends using the CIEDE2000 formula, which utilizes the concept of chroma and hue, providing a better correlation to the visual observations from the average observers. The CIEDE2000 color difference (ΔE_00_) is calculated using the following formula [18].

$$\Delta E_{00}\;=\;{\left[{\left(\frac{\Delta L}{K_{L}S_{L}}\right)}^{2}+{\left(\frac{\Delta C}{K_{C}S_{C}}\right)}^{2}+{\left(\frac{\Delta H}{K_{H}S_{H}}\right)}^{2}+R_{T}\left(\frac{\Delta C}{K_{C}S_{C}}\right)\left(\frac{\Delta H}{K_{H}S_{H}}\right)\right]}^{1/2}$$

where ΔL = |L peripheral − L central|, ΔC = |C peripheral − C central|, and ΔH = |H peripheral − H central| represent the changes in lightness, chroma, and hue for the samples, respectively, and R_T_ is a rotation function that accounts for the interaction between the chroma and hue differences in the blue region. Weighting functions, S_L_, S_C_, and S_H_, adjust the total color difference for variation in the location of the color difference, and the parametric factors, K_L_, K_C_, K_H_, are proposed as a way to control the changes in the magnitude of tolerance judgments and as a way to adjust for scaling of acceptability rather than perceptibility. Previous studies have already evaluated that the texture only affects lightness tolerances but not chroma or hue tolerances; therefore, the value of K_L_ = 2 was proposed [19].

### 2.3. Light Transmittance and Spectral Reflectance

Fifteen disk-shaped specimens (diameter = 35 mm and thickness = 0.5 mm) were made for light transmittance and spectral reflectance measurements using the tested flowable resin composites—OCF, GLF, and SUF. All of the specimens were then covered with polyethylene films. These specimens were light-cured for 30 s on both sides with an LED curing light (PenCure 2000, Morita, Tokyo, Japan), ranging from 660 to 760 mW/cm^2^, and then the polyethylene films were removed. The specimens were then immersed in the water for 24 h at 37 °C. After water storage, all of the specimens’ reflectance and transmittance were measured with a black background using a spectrophotometer (TC-1800MKII, Densyoku, Tokyo, Japan) under D65 illumination. The spectrophotometer was calibrated to a white standard before each series of measurements. Values of the spectral reflectance and transmittance for wavelengths were obtained from 380 nm to 780 nm, with a focus measuring aperture of 1° at the center of each disc. The data were averaged after five repeated measurements were performed without placement. An illustrated flowchart of the preparation of the specimen for light transmittance and reflectance is shown in Figure 1.

### 2.4. Statistical Analysis

The normality and homogeneity were evaluated using the Shapiro–Wilk and Levene tests. Kruskal–Wallis analysis was performed to analyze ΔE_00_, ΔL, ΔC, and ΔH at both periods (immediate and 28 days), whereas the Wilcoxon tests were used to compare the results between those measurement periods. The level of significance was set at 0.05. All of the statistical analyses were performed using a standard statistical software package (SPSS 26.0, Chicago, IL, USA). 

## 3. Results

### 3.1. Color Measurement 

**Figure 2:**
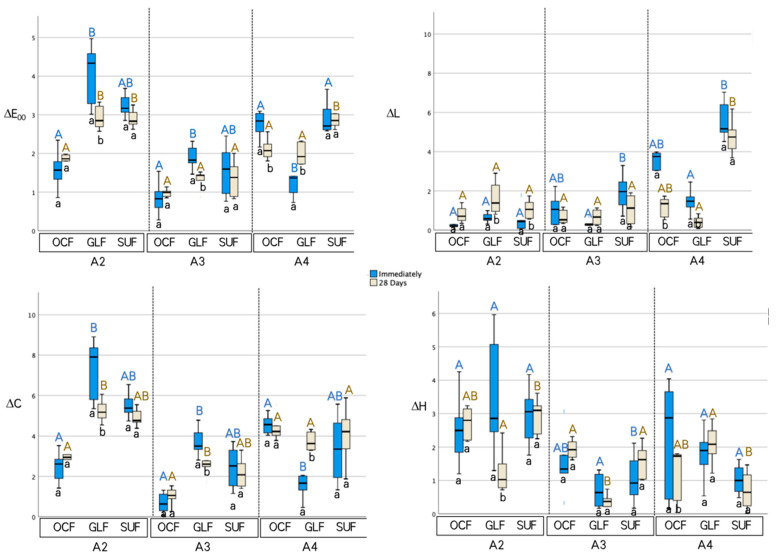
Box-and-whisker plots for the ΔE_00_, ΔL, ΔC, and ΔH values of three flowable resin composites at A2, A3, and A4 shade immediately and after 28 days of storage. Different upper-case letters show statistical differences between three different materials. Different lower-case letters show a statistical difference between different aging procedures.

The ΔE_00_, ΔL, ΔC, and ΔH values of three flowable resin composites at A2, A3, and A4 shades were plotted using a box-and-whisker plot with the medians (Q.50) and lower (Q.25) and upper quartiles (Q.75) immediately and after 28 days of storage and are shown in Figure 2. The closer the Y-axial is to zero, the better the color matching (ΔE_00_), lightness matching (ΔL), chroma matching (ΔC), and hue matching (ΔH) of the tested flowable resin composite.

Significant differences were observed in the A2 shade of ΔE_00_ between OCF and GLF both immediately and after 28 days of storage (*p* < 0.05). After 28 days of storage, OCF and SUF showed significant differences in the A2 shade. The A2 and A3 shades of OCF showed the lowest ΔE_00_ value compared with the other tested materials immediately after 28 days of storage. In the A4 shade, GLF was significantly lower than OCF and SUF immediately after color measurement (*p* < 0.05), whereas SUF showed significant differences between OCF and GLF after 28 days of storage (*p* < 0.05). There was a significant difference within each group in the different storage times for GLF in the A2, A3, and A4 shades and OCF in the A4 shade, while there was no significant difference in the different storage times for SUF in all of the shades. 

The ΔL of OCF, GLF, and SUF showed no significant differences between the groups immediately and after 28 days in the A2 shade. In the A3 shade, SUF was significantly higher than GLF immediately after storage, and both immediately and after storage in the A4 shade. There was a significant difference within the groups at different storage times for GLF and SUF in the A2 shade and OCF and GLF in the A4 shade.

The ΔC of OCF was significantly lower compared with GLF in the A2 and A3 shades both immediately and after 28 days of storage (*p* < 0.05), whereas in the A4 shade, GLF was significantly lower compared with OCF after immediate measurement (*p* < 0.05). There was a significant difference within the groups for different storage times of GLF in the A2, A3, and A4 shades.

The ΔH of GLF was significantly lower than SUF in the A2 shade after 28 days of storage. In the A3 shade, GLF was significantly lower than SUF after the immediate measurement and was significantly lower than OCF and SUF after 28 days of storage. In the A4 shade, SUF was significantly lower than GLF after 28 days of measurement (*p* < 0.05). There was a significant difference within each group at different storage times for GLF in the A2 shade and OCF in the A4 shade.

### 3.2. Spectral Behavior

**Figure 3:**
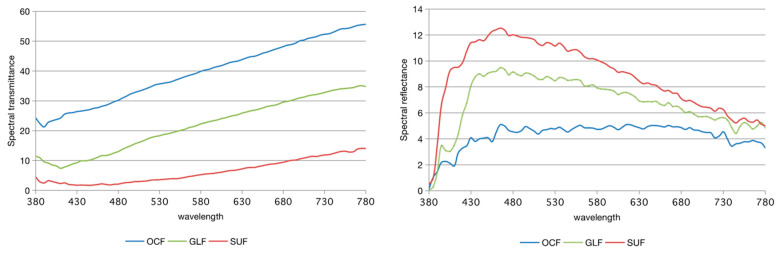
Distribution of spectral transmittance and spectral reflectance of each of the tested flowable resin composites.

The distribution of the spectral reflectance and spectral transmittance of each of the tested flowable resin composites are presented in Figure 3. The transmittance behavior of three tested FRCs showed a slight increase in wavelength of 480 nm to 780 nm. OCF showed the highest transmittance characteristic, followed by GLF and SUF. Similarly, the spectral reflectance curves showed a mountain-like shape curve of SUF and GLF, where the wavelength was between 380 nm and 500 nm. On the other hand, OCF showed a relatively stable reflectance curve in all of the wavelengths.

## 4. Discussion

Using the CIEDE2000 color-difference formula rather than the CIELAB formula when evaluating color difference provides more accurate indicators of human perception and acceptance across tooth colors [18,19,20]. Clinical instrumental color analysis should employ the CIEDE2000 color-difference formula rather than the ΔE^∗^ab formula [19]. Therefore, the CIEDE2000 formula was used in this present study. It would only be able to precisely measure perceptibility and acceptability thresholds through visual and instrumental approaches [21]. The 50:50% perceptibility threshold is reached when the color difference between comparable objects can be perceived by 50% of observers. The remaining 50% will not perceive any change. The 50:50% acceptability threshold is reached when half of the observers find the color difference acceptable. The other half find it unacceptable [18,21]. In dentistry, a perceptible color match is one in which the color difference is equal to or less than the perceptibility threshold, and an acceptable color match is one in which the color difference is equal to or less than the acceptability threshold [18]. Several studies have been conducted to determine the acceptability threshold (AT) and perceptibility threshold (PT) of color difference (ΔE_00_) [22]. According to previous reports, the 50:50% AT was determined to be 2.7, and the 50:50% PT was 1.2 for color difference [21]. Lightness, chroma, and hue are three elements that are used for obtaining an accurate color matching that corresponds to human eye perception [23]. The AT values of color coordinate differences for lightness, chroma, and hue have been previously reported, which are ΔL_AT_ = 2.92, ΔC_AT_ = 2.52, and ΔH_AT_ = 1.90, respectively [24]. These thresholds could lead to a valid and applicable formula for improving the modeling of tooth-colored aesthetic materials and, as a result, patient satisfaction [22,23,24].

The ΔE_00_ for OCF was lower than the AT in the A2 and A3 shades both immediately and after 28 days, and in the A4 shade after 28 days of storage. On the other hand, the ΔE_00_ value of GLF was below the AT in the A3 and A4 shades immediately and after 28 days. The A2 and A3 shades of OCF were demonstrated to be aesthetically color-matched, whereas GLF was shown in the A3 and A4 shades. ΔC demonstrated a similar tendency as ΔE_00_ for all materials. It can be assumed that the value of ΔE_00_ was mainly attributed to the ΔC values. Pecho et al. [25] evaluated the lightness, chroma, and hue differences in visual shade matching, and their results demonstrated that the chroma difference mainly determined the visual shade matching. The finding of the present study coincided with Pecho’s states. In the previous studies [16], the paste-type universal shade resin composite of Omnichroma showed a barely invisible color difference in the A2 and A3 shade resin composite molds, with an ΔE value of 0.8 ± 0.3 in the A2 shade and 1.7 ± 0.6 in the A3 shade. The flowable resin composite of OCF with the same uniform supra-nano size filler but dispersed filler distance also showed a similarly satisfied color matching in the A2 and A3 shades, which verified that structural color might work effectively with lower filling loading. Significant differences were observed in the shade matching and corresponding color parameters in the present study. Therefore, the first hypothesis was partially rejected. 

The color of a resin composite is influenced by the light scattering and absorption of the material and the material’s reflectivity and translucency [6,26]. The incident light in the resin composite is reflected by the resin matrix, filler, pigments, and background color and is then perceived as a specific color [27]. The interaction between optical scattering and absorption causes changes in color parameters due to the filtering effects of the translucent material over a background. The resin composite materials’ thickness and background reflectance can influence the color coordinates [27]. OCF, containing no pigment, showed a relatively uniform reflectance in the black background, mainly attributed to the reflection of filler particles, which is approximately 260 nm to induce structural color. Furthermore, OCF showed pronounced transmittance characteristics compared with its counterparts. Therefore, the authors speculate that the pronounced transmittance characteristics and the structural color may have a combined effect on the OCF resin composite’s shade matching. It has been reported that resin composite restorations have higher transmission properties and can effectively utilize the background color for color matching in shallow cavities [17]. In the present study, the high transmittance characteristic of the OCF resin composite allowed for the light reflection of the surrounding cavities, which profoundly affected the shallow cavities. As a result, this high transmittance characteristic would have a reverse effect in dark cavities because of the discriminative shade of the surroundings. Nevertheless, the reflection of the yellow-to-red color of the OCF resin composite was still insufficient to overcome the reverse effect of the high transmittance characteristics in the darker surroundings in order to meet the needs of the clinical standards.

Pigment contented GLF showed a relatively weak light transmittance and a mountain-like curve shape of reflectance at a wavelength ranging from 380 nm to 500 nm. The absorption of the specified wavelength was most likely due to the pigments present. The translucency of the resin composite was investigated by Kamishima et al. [28], who found opaque-shade resin composites could effectively minimize the effect of the dark background color of the oral cavity. In our study, the relatively opaque shade of GLF corresponded better with the darker cavities of the surroundings, owing to the filling resin composite’s apparent diffusivity, which resulted in a weaker color light production from the surroundings. The more pronounced scattering characteristic matched better with the dark part of the cavity in the A4 shade, whereas the color matching ability of the shallow part of the cavity in the A2 shade was unsatisfactory.

For color stability measurement, deionized distilled water was used as the test standard because saliva is a diluted fluid containing 99% water [29]. The color stability of the SUF resin composites was the best of all of the flowable resin composites evaluated, followed by OCF, and finally GLF. Water solubility could be attributed to discoloration by chemically degrading the filler–resin bond of the resin matrix [30]. Some previous studies concluded that water sorption and filler loading had a strong correlation [31,32]. A lower filler loading means that a higher resin matrix yields higher water sorption values, leading to discoloration [11]. This may explain why the highly filled SUF showed the most profound color stability among all of the tested flowable resin composites. Unlike OCFs, where the characteristic reflection of the wavelength is resistant to fading, GLF color comes mainly from their pigments. Hydrolysis changes in the chemical pigment may cause color differences after aging. Furthermore, the lower filler content of GLF may also be one of the factors contributing to its instability. Thus, the second hypothesis that tested the flowable resin composite’s color stability showed no significant difference and was rejected.

However, there were still some limitations to the experiment. The ESQ mold’s highest saturation and lowest lightness made the color matching of OCF more complex than its competitors. This could be another reason for the failure of the A4 shade of OCF. Color and spectral behaviors of esthetic resin composites would be more difficult to predict when infiltrating into different staining solutions rather than distilled water, and different polishing methods in clinical applications could also affect color parameters [33]. Further studies should be performed to evaluate the color matching and corresponding color parameters using natural human teeth. 

## 5. Conclusions

With the limitations of this study, it was concluded that (1) the OCF flowable resin composite showed better color matching with the surrounding shades of A2 and A3, while the GLF flowable resin composite showed better shade matching with the surrounding shade A4. (2) The OCF flowable resin composites showed relatively uniform reflectance and higher transmission properties in the shallow cavities. Therefore, the use of the OCF flowable resin composite allowed for a less complex procedure and resulted in a restoration that matched the surrounding tooth color, which is clinically acceptable.

## Figures and Tables

**Table 1:** Manufacturers, shades, filler contents, and monomers of paste-type composite for the molds and flowable composites for cavities.

	Materials	Manufacturer	Shades and Filler Content	Monomer
Paste-Type Composite (Mold)	Estelite Sigma Quick (ESQ)	Tokuyama Dental, Tokyo, Japan	A2, A3, A4 (82 wt%)	Bis-GMA, TEGDMA
	Gracefil Putty (GP)	GC Corporation, Tokyo, Japan	A2, A3, A4 (82 wt%)	Bis-MEPP, UDMA
	Filtek Supreme Ultra (FSU)	3M ESPE, St Paul, MN, USA	A2, A3, A4 (72.5% wt%)	Bis-EMA, Bis-GMA, UDMA
Flowable composite (Cavity)	OCF-001 (OCF)	Tokuyama Dental, Tokyo, Japan	Universal (71 wt%)	UDMA, Low viscosity monomer
	Gracefil LoFlo (GLF)	GC Corporation, Tokyo, Japan	Universal (69 wt%)	Bis-MEPP
	Supreme Ultra Flowable (SUF)	3M ESPE, St Paul, MN, USA	A3B (78.5 wt%)	Bis-GMA, TEGDMA

Bis-GMA, bisphenol A-glycidyl methacrylate; TEGDMA, triethylene glycol dimethacrylate; Bis-MEPP, bisphenole A ethoxylate dimethacrylate; UDMA, urethane dimethacrylate; Bis-EMA, bisphenol A ethoxylated dimethacrylate.

## Data Availability

All data are contained in the paper.
